# An early burst of cytokine production before the first cell division influences CD8 T cell differentiation

**DOI:** 10.1093/jimmun/vkaf239

**Published:** 2025-09-16

**Authors:** Shannon M Kahan, Jennifer T Ingram, Robert S Welner, Casey T Weaver, Laurie E Harrington, Allan J Zajac

**Affiliations:** Department of Microbiology, University of Alabama at Birmingham, Birmingham, AL 35294, United States; Department of Microbiology, University of Alabama at Birmingham, Birmingham, AL 35294, United States; Department of Medicine, University of Alabama at Birmingham, Birmingham, AL 35294, United States; Department of Pathology, University of Alabama at Birmingham, Birmingham, AL 35294, United States; Department of Cell, Developmental and Integrative Biology, University of Alabama at Birmingham, Birmingham, AL 35294, United States; Department of Microbiology, University of Alabama at Birmingham, Birmingham, AL 35294, United States

**Keywords:** cytokines, effector, memory, T cells

## Abstract

The differentiation of CD8 T cells into effector and memory populations is guided by a combination of antigenic, costimulatory, and cytokine signals. Here we show that, within 24 h of activating naïve CD8 T cells, populations emerge with divergent patterns of interleukin (IL)-2 and interferon (IFN)-γ synthesis. This rapid, dynamic, and heterogeneous burst of cytokine production manifests with every CD8 T cell specificity analyzed, is apparent in vivo and in vitro, and occurs prior to the first cell division. Nevertheless, how the intrinsic manufacture of distinct cytokines forecasts and influences the properties and fates of the producer cell itself are not well defined. We demonstrate that the initial cell intrinsic synthesis of IL-2 attenuates IL-2-dependent STAT5 signaling, but that this is not due to differences in the surface expression of the IL-2 receptor complex. The functionally discrete subsets are transcriptionally distinct and display differences in the expression of hallmark effector and memory associated genes. Using cytokine reporter systems, we reveal that these early functional differences are consequential for establishing fate biases and directing the gain of effector and memory T cell properties. The bifurcation between the abilities of IL-2-producing and non-producing subsets to elaborate STAT5 signaling is consistent with a model in which non-IL-2-producing CD8 T cells are more receptive to extrinsic IL-2 signals and preferentially contribute to the early surge of effector formation. Despite this, both IL-2-producing and non-producing CD8 T cells can go on to acquire memory traits, indicating that there is developmental diversity within each cytokine producing subset.

## Introduction

Effector and memory CD8 T cell subsets are vital contributors to the immune mediated control of infections and tumors. The formation of these populations is initiated when naïve CD8 T cells engage their cognate antigen bound to a major histocompatibility class-I molecule, which triggers T cell receptor signaling. The subsequent differentiation of effector and memory populations is not only instigated by antigenic activation but enforced by costimulatory molecules, cytokines, and nutrients.^1^^,^^2^ This results in biochemical and transcriptional modifications that allow rapid expansion and the gain of effector properties, and in the imprinting of functional capabilities and longevity. Accordingly, the collective signals that CD8 T cells encounter following activation can dictate the magnitude and protective efficacy of the response, as well as the subsequent establishment of the memory pool.

It has been shown that a 2 h activation period is sufficient to stimulate CD8 T cell clonal expansion and differentiation whereas slightly longer activation can drive both proliferation and the more complete acquisition of effector and memory traits.^3–5^ Thus, stimulation of naïve CD8 T cells for 20 to 24 h can elicit multiple rounds of cell division and the development of effector and memory features even if the activating antigen is withdrawn. Notably, the “autopilot” expansion that occurs following transient activation requires the presence of exogenous IL-2.^4^ The endowment of effector and memory traits has also been reported to be established upon CD8 T cells’ first division following activation. Asymmetric cell division can occur at cytokinesis, with the daughter T cell proximal to the activating antigen-presenting cell preferentially gaining effector traits and the distal daughter cell predisposed to attaining memory properties.^6–8^ Accordingly, the development of effector and memory traits by CD8 T cells is steered by signals received during the very early activation phase.

Interleukin (IL)-2 is a cytokine that serves as a critical differentiation factor that influences the formation of effector and memory CD8 T cells.^9^^,^^10^ Stimulation with IL-2 has been shown to act in conjunction with antigen-dependent T cell receptor (TCR)-mediated signals to promote the development of effector cells and to drive terminal differentiation.^11^^,^^12^ This is achieved in part by the upregulation of eomesodermin and perforin as well as by the repression of Bcl6 and CD127.^12^ Conversely, IL-2 signals have also been shown to promote memory properties.^13–15^ Activated CD8 T cells can manufacture IL-2, allowing them to serve as an autonomous source of this cytokine; however, paracrine signals, potentially from other CD8 T cells, CD4 T cells or dendritic cells, have also been implicated in regulating responses.^13^^,^^15–17^ Nevertheless, how the intrinsic manufacture of IL-2 influences the properties and fates of the producer cell itself are not well defined.

Here, we set out to analytically deconstruct whether and how the very rapid induction of cytokine synthesis, which occurs as naïve CD8 T cells first become activated, determines the properties and differentiation state of the ensuing response. Using cytokine reporter systems,^17–19^ we dissected whether the fates of IL-2 producing and non-producing CD8 T cells diverged prior to the first cell division following activation. We discovered that the synthesis of IL-2 limited both expansion as well as the gain of effector traits by the producer cell, and this was accompanied with diminished STAT5 phosphorylation. Conversely, naïve CD8 T cells that did not initiate IL-2 synthesis immediately following activation were biased to increase in number and acquire effector properties. Nevertheless, both IL-2-producing and non-producing CD8 T cells could establish a memory pool. Thus, although memory formation can proceed independently of endogenous IL-2 production, the early cell intrinsic manufacture of IL-2 attenuates the initial surge of effector differentiation.

## Materials and methods

### Mice

B6.Cg-*Thy1^a^*/Cy Tg(TcraTcrb)8Rest/J (pmel-1) mice, which express an H2D^b^-restricted T cell receptor (TCR) specific for the premelanosome gp100_25-33_ epitope,^20^ and C57BL/6J-*Ptprc^em6Lutzy^*/J CD45.1 (JAXBoy) mice were purchased from the Jackson Laboratory (Bar Harbor, Maine). P14 transgenic mice that express a TCR specific for the H2D^b^-restricted LCMV-GP33-41 epitope^21^ were originally kindly provided by Dr Rafi Ahmed (Emory University). P14 TCR transgenic mice homozygous for the knockin *Ifng*^tm1(Thy1)Weav^ and *IL2^tm1(eGFP)Weav^* cytokine reporters (P14 IFN-γ.Thy1.1 IL-2.GFP) and the CD45.1^+^CD45.2^+^ heterozygous cytokine reporter recipients have been previously described.^17^ C57BL/6 Tg (TcraTcrb)1100Mjb/J (OT-I) mice, which express TCR transgenes specific for the H2K^b^-restricted ovalbumin 257-264 epitope,^22^ were provided by Dr Frances Lund (University of Alabama at Birmingham [UAB]). Mice were bred and maintained in accredited facilities at UAB. All procedures were approved by the UAB Institutional Animal Care and Use Committee. Both male and female mice were used.

### Verification of TCR transgene expression and naïve phenotypes

Expression of the P14, OT-I, and pmel-1 TCR transgenes was verified by flow cytometric analyses of splenocytes stained with anti-CD8α antibodies (53-6.7). P14 cells were costained with anti-TCR Vα2 (B20.1) and Vβ8.1/8.2 antibodies (KJ16-133.18). OT-I cells were co-stained with anti-TCR Vα2 antibodies together with H2K^b^(OVA) tetramers. pmel-1 TCR transgenic cells were stained with anti-TCR Vα2 and anti-TCR Vβ13 (MR12-3) antibodies. Note that pmel-1 TCR transgenic CD8 T cells are TCR Vα1+, Vβ13+; however, antibodies against murine Vα1 were not available, thus anti-Vα2 antibodies were included as a negative control.^20^

The naïve status of splenocytes from P14, OT-I, and pmel-1 TCR transgenic mice was verified by staining with anti-CD8α, anti-CD44 (IM7) and anti-CD62L (MEL-14) antibodies.

### In vitro activation

Suspensions of murine splenocytes were prepared in RPMI-1640 supplemented with 10% fetal bovine serum, 2 mM L-glutamine, 100 U/ml penicillin, 100 μg/ml streptomycin, and 50 μM 2-mercaptoethanol (R10). Cells were plated at a concentration of 1.5 × 10^6^ cells/ml. P14, OT-I, and pmel-1 splenocytes were activated with 1 μg/ml of their cognate peptide antigen KAVYNFATM,^21^ SIINFEKL,^22^ and EGSRNQDWL,^20^ respectively (Bio-synthesis, Lewisville, TX). Brefeldin A (GolgiPlug; BD Biosciences, San Jose, California) was added for the last hour of the culture period. Cells were then harvested and stained for CD8α (53-6.7), CD25 (PC61), CD69 (H1.2F3) and, in some experiments, Thy1.1 (OX-7) and Thy1.2 (30-H12) followed by intracellular staining for IFN-γ (XMG1.2) and IL-2 (JES6-5H4). Splenocytes from P14 IFN-γ.Thy1.1 IL-2.GFP mice were activated for 20 h with 1 μg/ml GP33 peptide without the addition of Brefeldin A. After stimulation cells were washed and stained for CD8-PB (53-6.7), CD69-APCe780 (H1.2F3), CD25-PerCP-Cy5.5 (PC61), Thy1.1-PE-Cy7 (OX-7), CD122-PE (TM-b1), and CD132-APC (TUGm2). Intracellular staining was performed using the BD Cytofix/Cytoperm Fixation/Permeabilization kit (BD Biosciences) in accordance with the manufacturer’s instructions. Samples were acquired using an LSRII flow cytometer (BD Biosciences) and analyzed using FlowJo software (BD Life Sciences). Unless otherwise stated antibodies were purchased from Biolegend or Thermo Fisher Scientific (eBioscience, Invitrogen).

### In vivo activation

CD8 T cells were enriched from the spleens of P14 mice using the CD8a^+^ T Cell Isolation Kit (Miltenyi Biotec, San Diego, California) with the addition of 0.001 μg anti-CD44 biotin (IM7) per million cells during the antibody staining step of the purification procedure. Enriched P14 CD8 T cells (7.5 × 10^5^) were adoptively transferred into CD45.1 JAXBoy recipient mice by retro orbital injection. These recipient mice were infected the next day with 2 × 10^6^ pfu LCMV-Armstrong i.p. At 14 or 24 h following infection spleens were explanted and splenocytes were prepared by digestion with 2 mg/ml collagenase D (Roche, Indianapolis, Indiana) and 0.03 mg/ml DNase I (Sigma-Aldrich, St Louis, Missouri) for 30 min at 37 °C and then further disrupted prior to red blood cell lysis and washing. Spleens were harvested and processed in media and buffers containing 10 μg/ml Brefeldin A. Cell suspensions were then treated with LIVE/DEAD Fixable Aqua dye (Invitrogen, Carlsbad, CA) and stained with anti-CD8α-PE-Cy7 (53-6.7), anti-CD69-PE (H1.2F3), anti-CD25-FITC (PC61), anti-CD45.1-APCe780 (A20), anti-CD45.2-PerCP (104), and then intracellular staining for IFN-γ-PB (XMG1.2) and anti-IL-2-APC (JES6-5H4) was performed after fixation with 4% formaldehyde (Fisher Scientific, Fair Lawn, New Jersey) in phosphate-buffered saline (PBS) for 20 min at room temperature followed by permeabilization using 0.1% saponin (S7900, Sigma-Aldrich) in PBS with 1% FCS and 0.1% NaN_3_ for 10 min at room temperature as described by Pala et al.^23^ Samples were acquired using an LSRII flow cytometer (BD Biosciences) and analyzed using FlowJo software (BD Life Sciences).

### CFSE analyses

Splenocytes from P14 mice were labeled with 1 μM carboxyfluorescein succinimidyl ester (CFSE; Invitrogen) in PBS for 10 min at 37 °C. Labeling was quenched by the addition of FCS and cells washed 2 times with R10. The CFSE labeled splenocytes (1 × 10^7^ cells/ml) were then cultured in a 96 well plate together with 1 μg/ml GP33 peptide. A 1:1000 dilution of Brefeldin A (GolgiPlug; BD Biosciences) was added for the last hour of culture. Cells were harvested after 0, 12, 24, and 48 h of activation and stained for CD8-PE-Cy7 (53-6.7), CD69-PE (H1.2F3), CD25-APCe780 (PC61) followed by intracellular staining for IFN-γ-e450 (XMG1.2) and IL-2-APC (JES6-5H4) using the BD Cytofix/Cytoperm Fixation/Permeabilization kit (BD Biosciences).

### Cell sorting and STAT5/pSTAT5 staining

Splenocytes from P14 IFN-γ.Thy1.1 IL-2.GFP mice were activated in vitro by addition of the GP33 peptide (1 μg/ml). In certain instances IL-2 blocking antibodies (100 μg/ml anti-CD25 (PC61.5.3), 100 μg/ml anti-IL-2 (JES6-1A12), and 100 μg/ml anti-IL-2 (S4B6-1); BioXcell, Lebanon, New Hampshire) were added for either the last hour or entire 20 h duration of the culture period. Following activation formaldehyde (Fisher Scientific Cat no. F79-1) was added directly into the cultures to a final concentration of 0.7%. After an additional 15 min, cell were washed twice with PBS + 0.5% BSA prior to the addition of a surface staining cocktail. Splenocytes were stained using CD8-PB (53-6.7) and Thy1.1-PE-CY7 (OX-7) anitbodies. The IL-2 (GFP)^+^, IL-2 (GFP)^+^ IFN-γ (Thy1.1)^+^, IFN-γ (Thy1.1)^+^, and IL-2 (GFP)^-^ IFN-γ (Thy1.1)^-^ subsets of CD8 T cells were subsequently isolated by cell sorting using a BD FACS ARIA II cell sorter. Following sorting each purified population was fixed with 1.5% paraformaldehyde in PBS and permeabilized with 100% methanol for 30 min. Cells were then washed in PBS + 0.5% BSA, and stained with anti-STAT5A-PE (C-6; Santa Cruz Biotechnology) and anti-pSTAT5a-A647 (47/Stat5(pY694); BD Biosciences) antibodies in PBS with 0.5% BSA.^17^^,^^24^

### RNA preparation and sequencing

Splenocytes from naive male and female P14 IFN-γ.Thy1.1 IL-2.GFP mice were stimulated with the LCMV GP33 peptide epitope (1 μg/ml) for 20 h. Stimulated and control unstimulated splenocytes were then stained with CD8-PB (53-6.7), CD69-PE (H1.2F3), and Thy1.1-PE-Cy7 (OX-7). Approximately 500,000 P14 CD69^+^CD8^+^ Thy1.1^+^ GFP^+^ (IFN-γ^+^IL-2^+^), Thy1.1^+^ GFP^-^ (IFN-γ^+^IL-2^-^), Thy1.1^-^ GFP^+^ (IFN-γ^-^IL-2^+^), Thy1.1^-^ GFP^−^ (IFN-γ^-^IL-2^-^) cells, as well as ∼300,000 control unstimulated P14 IFN-γ.Thy1.1 IL-2.GFP CD8^+^ T cells, were then sorted directly into TRIzol LS (Invitrogen) using a BD FACS Aria IIIu cell sorter. RNA was isolated using a Direct-zol RNA isolation kit (Zymo Research, Irvine, California).

RNA samples were submitted to the UAB Heflin Center for Genomic Science (Birmingham, Alabama) for quality assessment, RNA sequencing, and analyses. RNA quality was assessed using an Agilent 2100 Bioanalyzer. Samples with an RNA integrity number (RIN) of ≥7.0 was used for sequencing library preparation. RNA passing quality control was converted to a sequencing ready library using the NEBNext Ultra II Directional RNA library kit with polyA selection as per the manufacturer’s instructions (NEB, Ipswich, Massachusetts). Briefly, two rounds of poly A selection were performed on total RNA with paramagnetic oligo dT beads. The purified mRNA was fragmented with heat and cations and converted to cDNA with a mixture of random primers for first strand synthesis followed by standard second strand synthesis. The resulting molecules were ligated to a universal adaptor. The Illumina sequences and the unique index information were added via PCR. The resulting cDNA libraries were quantitated using qPCR in a Roche LightCycler 480 with the Kapa Biosystems kit for Illumina library quantitation (Kapa Biosystems, Woburn, Massachusetts) prior to cluster generation. RNA-sequencing was performed on the Illumina NextSeq500 as described by the manufacturer (Illumina Inc., San Diego, California). The RNA sequencing analysis generated in these studies can be found in the Gene Expression Omnibus under accession number GSE294688.

### RNA-sequence data analyses

Raw sequencing data were processed using FastQC for quality control and trimmed using Cutadapt. Sequenced reads were aligned to GRCm38 for mouse using STAR aligner. Gene expression quantification was performed with featureCounts. Differential expression analysis was conducted using DESeq2, applying a threshold of adjusted *P*-value <0.05 and log2 fold change to define differentially expressed genes (DEGs). Principal component analysis (PCA) was performed using PCAtools to visualize the variance in gene expression profiles among different samples after removing the shared DEGs as compared to naïve CD8 T cells. The top genes were selected based on their supervised variance. Bubble plot analysis utilized ggplot2, where transcripts associated with effector and memory T cell formation, function, and maintenance were selected based on literature for visualization.^25–30^

### Cell sorting and adoptive transfers without infection

Splenocytes from P14 IFN-γ.Thy1.1 IL-2.GFP reporter mice were activated as described for the pSTAT5 staining experiments with and without blocking IL-2 signals for the 20 h activation period. Cells were harvested, washed, and stained with CD8-PB (53-6.7), CD69-PE (H1.2F3), and Thy1.1-PE-Cy7 (OX-7). IL-2 (GFP)^+^ and IL-2 (GFP)^-^ CD69^+^ CD8^+^ T cell subsets were then isolated by cell sorting using a BD FACS Aria IIIu instrument. Normalized numbers of each cell population (1 × 10^6^ cells) were transferred by i.v. injection into allelically marked CD45.1^+^CD45.2^+^ heterozygous cytokine reporter recipients mice. At 4 d following cell transfer splenic donor CD45.2 populations were quantitated and their expression of CD62L, Ki-67, T-bet, and granzyme B (GzmB) analyzed. Cells were stained with anti-CD8-PB or PE-Cy7 (53-6.7), anti-CD45.1-APCe780 (A20), anti-CD45.2-PerCP-Cy5.5 (104), anti-CD62L-PE (MEL-14), anti-Ki-67-PB (SolA15), anti-T-bet-APC (4B10), and anti-granzyme B-PE (GB12) antibodies. Intracellular staining was performed using the BD Cytofix/Cytoperm Fixation/Permeabilization Kit (BD Biosciences) and intranuclear staining was performed using the eBioscience Foxp3/Transcription Factor Staining Buffer Set (Invitrogen).

### Cell sorting and transfers with infection

Splenocytes from naive P14 IFN-γ.Thy1.1 IL-2.GFP mice were prepared and 3 × 10^7^ cells were seeded into individual wells of 6 well cluster plates (Corning, Corning, New York) in a final volume of 4 ml R10. Cells were activated by addition of LCMV GP33 peptide (1 μg/ml) for 20 h and then washed and stained with CD8-PB (53-6.7) and CD25-PE (PC61) in PBS, 1% FCS. GFP^+^ (IL-2^+^) and GFP^-^ (IL-2^-^) CD25^+^CD8 T cells were sorted using a FACS Aria II instrument (BD Biosciences) to a purity of >90% and collected into RPMI-1640 supplemented with 20% FCS. The sorted cells were washed and 2.5 × 10^5^ cells of either population were adoptively transferred into CD45.1^+^CD45.2^+^ mice that are heterozygous for both IFN-γ.Thy1.1 and IL-2.GFP reporters. One day later recipient mice were infected with 10^4^ pfu LCMV-Arm ip. At 3.5 or 32 d post-infection splenocytes were harvested from the recipient mice and the CD45.2 donor populations analyzed. Cells were stained with combinations of anti-CD8-PB (53-6.7), anti-CD45.1-APCe780 (A20), anti-CD45.2-PerCP-Cy5.5 (104), anti-CD62L-PE-Cy7 (MEL-14), anti-CD127-PE (A7R34), anti-KLRG1-APC (2F1), anti-CD27-PE-Cy7 (LG.7F9), and anti-CD43-A647 (1B11) antibodies. Additionally, aliquots of splenocytes were activated in vitro in the presence of 1 μg/ml GP33 peptide together with Brefeldin A for 5 h and subsequently intracellular staining with anti-IFN-γ-PB (XMG1.2) and anti-IL-2-APC (JES6-5H4) was performed using the BD Cytofix/Cytoperm Fixation/Permeabilization Kit (BD Biosciences). Samples were acquired using a BD FACSymphony A1 instrument (BD Biosciences).

### Statistical analysis

Two-tailed unpaired t tests were used to determine statistical significance between CD8 T cells activated in vivo following LCMV infection and also following adoptive transfer into recipients that were subsequently infected. Comparisons of 3 or more experimental groups were determined using 1- or 2-way analysis of variance (ANOVA). *P* values are defined as *P < 0.05, **P < 0.01, ***P < 0.001, and ****P < 0.0001.

## Results

### Naïve CD8 T cells undergo a rapid burst of cytokine synthesis following activation

**Figure 1.**
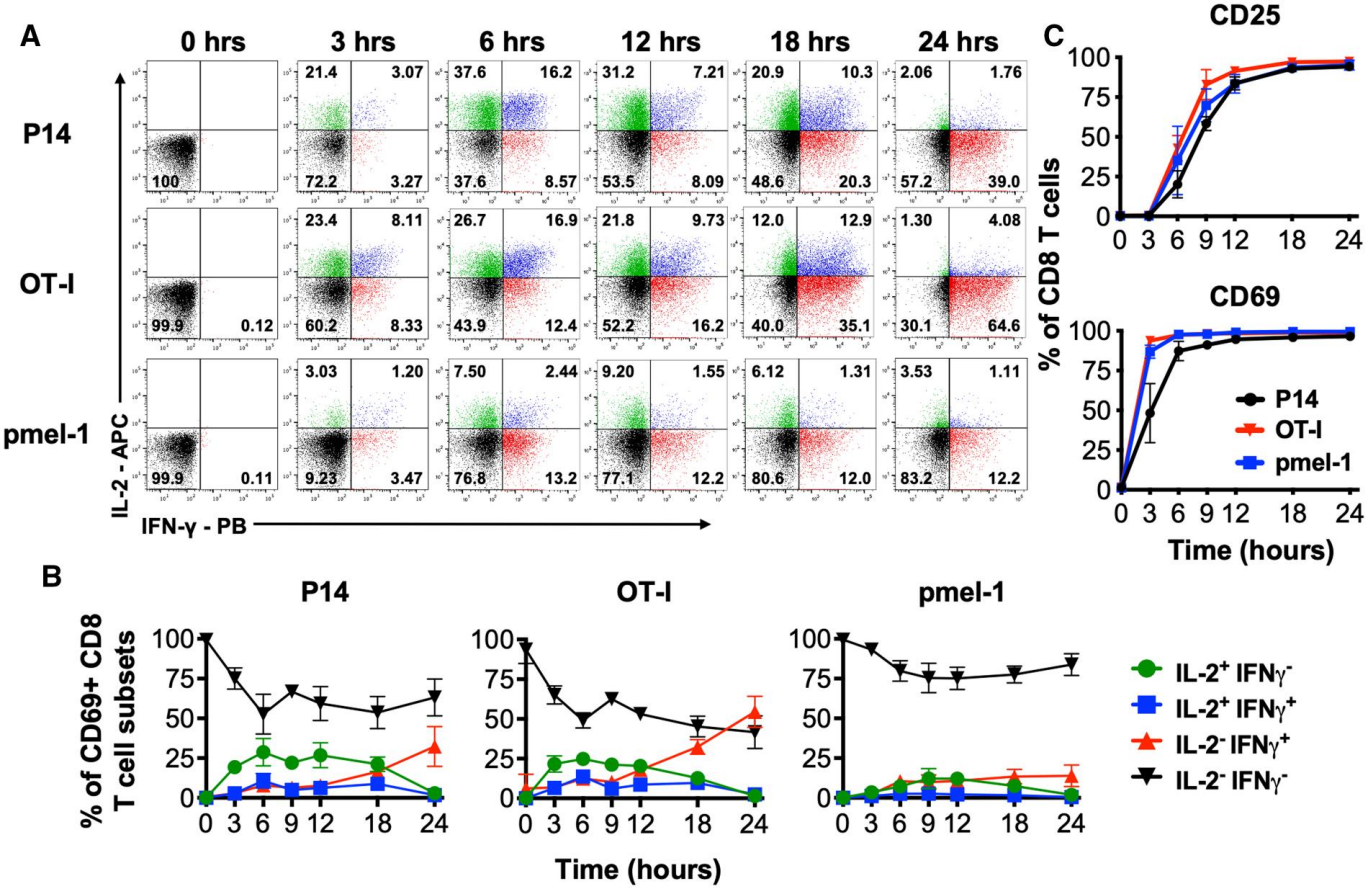
Naive CD8 T cells undergo a discordant burst of cytokine production upon activation. Splenocytes from the indicated naïve TCR transgenic mice were activated in vitro with their cognate peptide antigen and IL-2 and IFN-γ production as well as the upregulation of CD25 and CD69 assessed at the indicated time points. (A) Representative flow cytometry plots show IL-2 and IFN-γ intracellular staining profiles. Gated CD8 T cells are shown for the 0 hrs specimens. All other time points show gated CD69^+^ CD8 T cells. (B) Graphs show the proportions of the distinct cytokine producing populations over time. (C) Kinetics of CD25 and CD69 upregulation. Error bars show SD. Representative or composite data are shown from 3 to 7 independent experiments analyzing a total of 5 male and 3 female P14, 2 male and 2 female OT-I, and 4 male and 3 female pmel-1 mice.

To investigate whether the induction of cytokine synthesis influences CD8 T cell differentiation, we first defined the patterns of IL-2 and IFN-γ production that occur following activation in vitro (Fig. 1). For these analyses splenocytes from viral (P14),^21^ xeno (OT-I),^22^ or tumor (pmel)^20^ antigen-specific MHC class I-restricted TCR-transgenic mice were used. We verified that the vast majority of the CD8 T cells (∼86% to 96%) expressed the expected TCR transgenes and/or stained positive with the appropriate MHC tetramer (Fig. S1B). Note that pmel-1 TCR transgenic CD8 T cells are TCR Vα1+, Vβ13+; however, antibodies against murine Vα1 were not available, thus anti-Vα2 antibodies were included as a negative control. In addition, ∼84% to 94% of the CD8 T cells were CD44^lo^CD62L^hi^, confirming their naïve phenotype (Fig. S1C, D). Therefore, almost all the CD8 T cells analyzed expressed the expected TCR transgenes and were naive.

Activation of the TCR transgenic populations following activation with their cognate peptide antigen was assessed over a 24 h time span and the manufacture of IL-2 and IFN-γ determined (Fig. 1, Fig. S1A, E). The induction of IL-2 synthesis was rapid and detectable as early as 3 h following activation, and the patterns of cytokine production were diverse and dynamic (Fig. 1A and B, Fig. S1E). Over the 24 h activation period the initial burst of IL-2 synthesis subsided and IFN-γ became the predominant cytokine produced at later time points. Subsets that co-manufactured IL-2 and IFN-γ also initially formed but then declined. Further comparisons of the early cytokine production profiles of the 3 TCR transgenic populations showed that a lower fraction of pmel-1 CD8 T cells manufacture IL-2 and/or IFN-γ, when compared with P14 and OT-I CD8 T cells (Fig. S1E and Table S1). Moreover, as the responses progressed the proportion of IFN-γ^+^ CD8 T cells increased, and a hierarchy emerged, with a greater fraction of OT-I cells synthesizing IFN-γ and the lowest proportion of IFN-γ producers detected with pmel-1 CD8 T cells. Notably, even though a substantial fraction of CD8 T cells failed to manufacture detectable levels of either cytokine, all the cells displayed hallmarks of activation as by 12 h 83.3% to 91.4% and 94.6% to 98.4% of the stimulated cells upregulated expression of CD25 and CD69, respectively (Fig. 1C).

**Figure 2.**
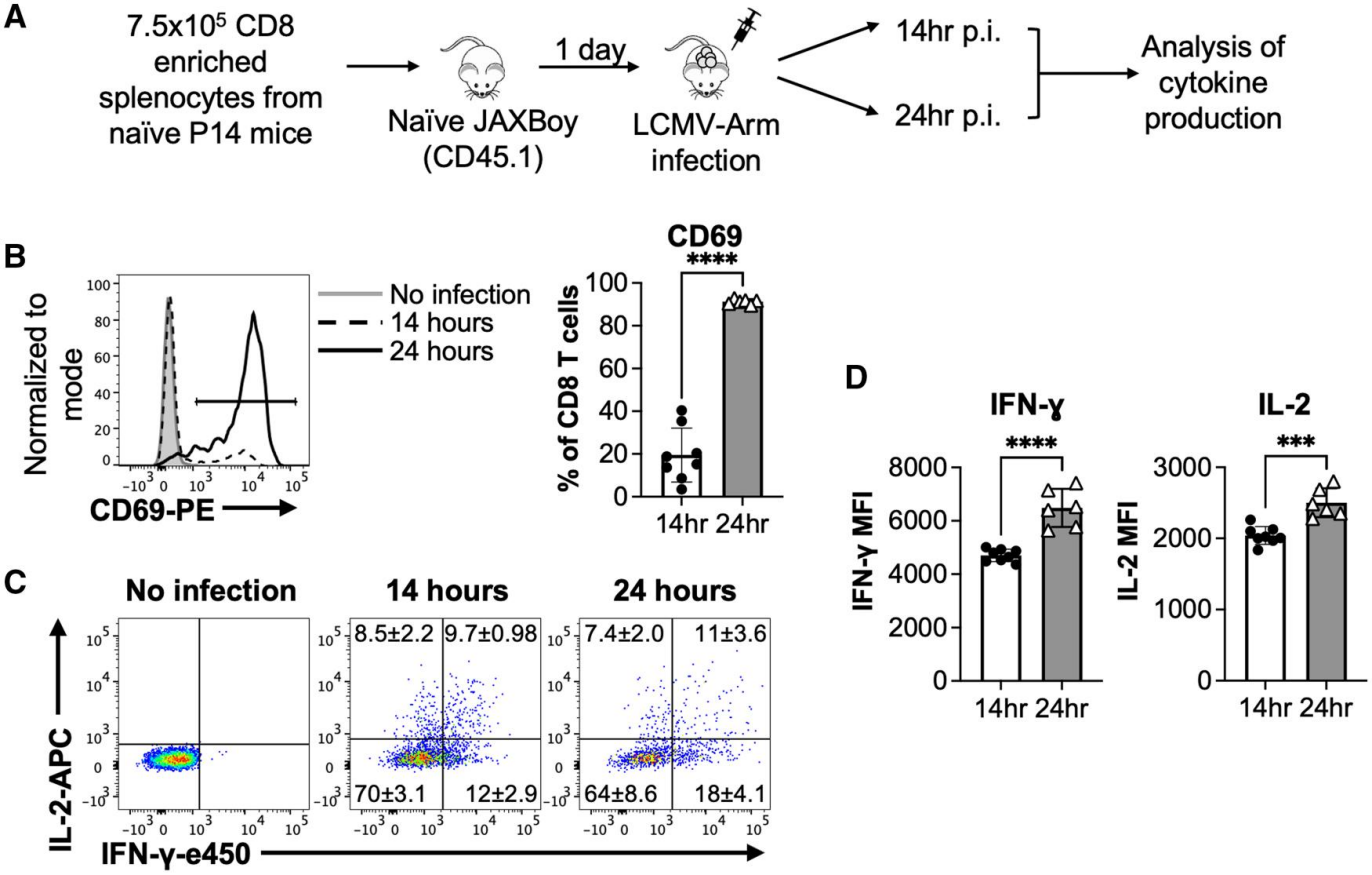
Virus-specific CD8 T cells undergo a heterogeneous burst of cytokine synthesis within hours of in vivo infection. (A) Experimental design. 7.5 × 10^5^ P14 CD8 T cells were adoptively transferred into allelically marked recipients that were infected with LCMV-Arm 1 d later. (B) Representative histograms and bar graph of cumulative data show the induction of CD69 expression by the donor P14 CD8 T cells at 14 and 24 h following infection. (C) Representative flow cytometry plots show IFN-γ and IL-2 production by donor CD8 T cells from control mice or gated CD69^+^ CD8 T cells at 14 and 24 h following infection. (D) Shows the MFI of either the IFN-γ or IL-2 cytokine producing contingent of donor CD69^+^ CD8 T cells. Bar graphs show means ± SD with significance calculated using an unpaired 2-tailed *t* test. *P < 0.05, **P < 0.01, ***P < 0.001, and ****P < 0.0001. Representative or cumulative results are shown from 2 independent experiments analyzing a total of 8 and 6 male recipients at the 14 h and 24 h time points, respectively.

To ascertain how naïve lymphocytic choriomeningitis virus (LCMV)-specific P14 CD8 T cells respond during the very earliest stages of infection, we conducted adoptive transfer experiments (Fig. 2A, Fig. S2A). By 14 h following infection ∼20% of the donor P14 CD8 T cells upregulated CD69 (Fig. 2B) and 4 distinct populations of cytokine producing cells became detectable (Fig. 2C). By 24 h following infection over 90% of the donor cells upregulated CD69, and cytokine production levels amplified, as indicated by increases in MFI (Fig. 2D). Thus, marked disparities in the initial and rapid burst of IL-2 and IFN-γ synthesis manifest as naïve CD8 T cells become activated both in vitro and in vivo.

### CD8 T cell functional diversification occurs prior to the first cell division

**Figure 3.**
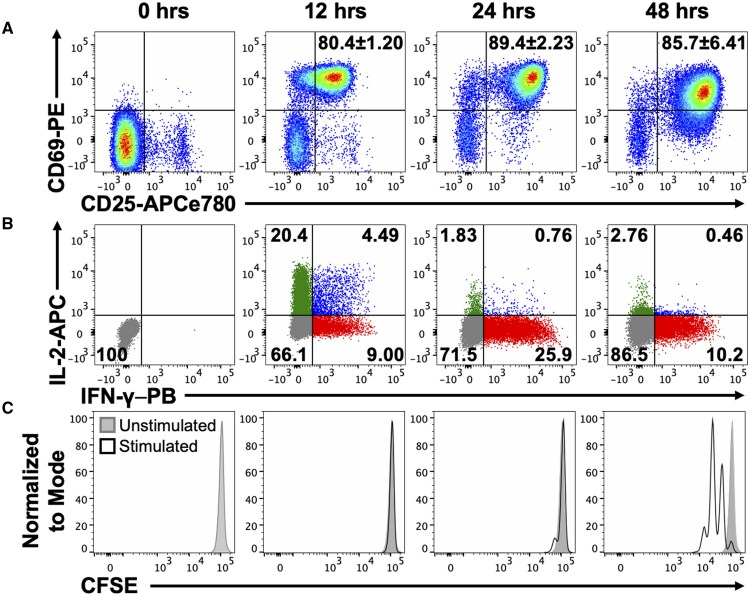
IL-2 and IFN-γ synthesis by newly activated CD8 T cells occurs prior to cell division. Representative plots show the induction of (A) CD69 and CD25 expression, (B) IL-2 and IFN-γ synthesis, and (C) shows the diminution of CFSE fluorescence over time following activation of naïve P14 splenocytes. Gated CD8 T cells are shown in A. Gated CD69+ CD8 T cells are shown in B and C, with the exception of the 0 h specimens and unstimulated controls, which show bulk CD8 T cells. Representative plots are shown from 3 independent experiments analyzing a total of 3 male mice.

Prior studies have shown that effector and memory CD8 T cell differentiation can be imprinted as the cells first divide following activation.^6–8^ Thus, we evaluated whether the distinct patterns of early cytokine production by CD8 T cells were associated with cell division. CFSE labeled splenocytes from naïve P14 TCR transgenic mice were stimulated in vitro with the GP33 antigenic peptide, and activation, cytokine synthesis, and proliferation were monitored over 48 h (Fig. 3). As expected, marked upregulation of CD25 and CD69 occurred by 12 h following activation (Fig. 3A), and heterogeneous populations of IL-2 and IFN-γ producing CD8 T cells emerged well within the first 24 h of stimulation (Fig. 3B). Nevertheless, populations that had undergone division were not detected until 48 h (Fig. 3C). Thus, the rapid and dynamic burst of cytokine synthesis is clearly apparent hours prior to the first cell division.

### Recently activated CD8 T cells with diverse cytokine production profiles express similar surface levels of the IL-2 receptor

**Figure 4.**
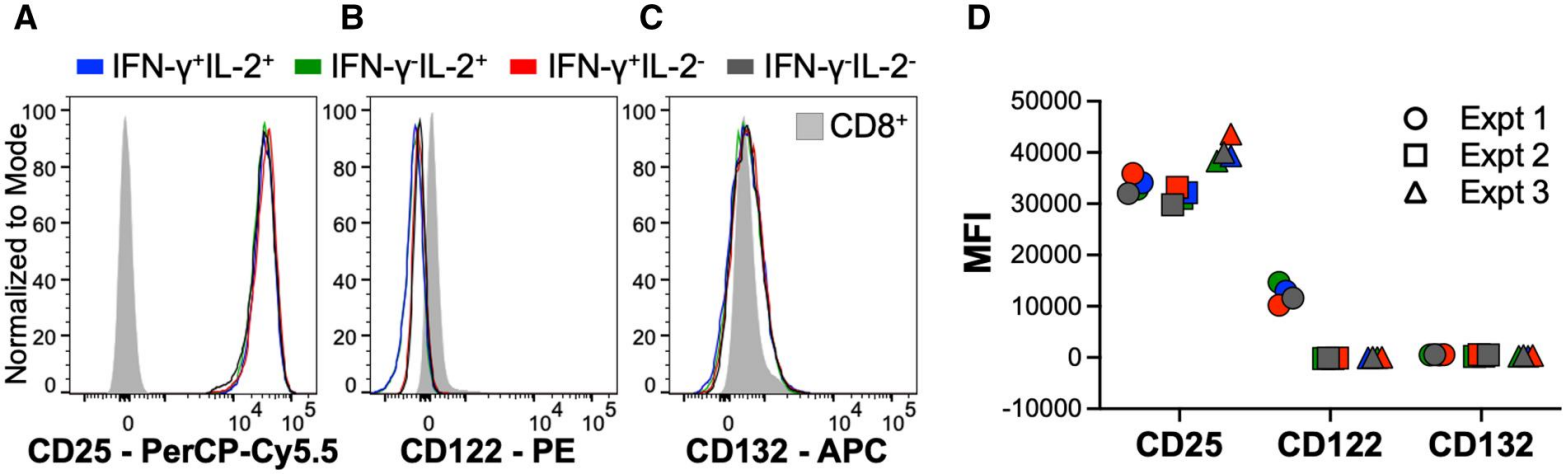
Recently activated CD8 T cells with diverse cytokine production profiles express similar surface levels of the IL-2 receptor. Splenocytes from P14 cytokine reporter mice were activated in vitro with the GP33 antigenic peptide for 20 h. Representative histograms show the expression of (A) CD25, (B) CD122, and (C) CD132 by the indicated functionally distinct CD8 T cell subsets. (D) Graphical representation of the results from three separate experiments. Representative data are shown from 3 independent experiments analyzing separate pools of spleens from 7 female, 6 female, and 6 male mice.

IL-2 signals are a driver of effector and memory CD8 T cell formation.^9–12^ Thus, given the differences in the patterns of early cytokine synthesis we evaluated the levels of IL-2 receptor expression by the distinct subsets of IL-2 and IFN-γ producing CD8 T cells (Fig. 4). To accomplish this we used P14 IFN-γ.Thy1.1 IL-2.GFP cytokine reporter mice, in which IL-2 and IFN-γ expressing cells are identifiable by the upregulation of GFP and Thy1.1, respectively.^17^ Splenocytes were activated in vitro with the GP33 antigenic peptide for 20 h, which is during the initial cytokine production period but before they undergo division. Each of the 4 distinct populations (IL-2^+^IFN-γ^-^, IL-2^+^IFN-γ^+^, IL-2^-^IFN-γ^+^, and IL-2^-^IFN-γ^-^) expressed similar surface levels of the IL-2 receptor subunits CD25 (IL-2Rα) (Fig. 4A and D), CD122 (IL-2Rβ) (Fig. 4B and D), and CD132 (IL-2Rγ) (Fig. 4C and D).

### IL-2-dependent STAT5 phosphorylation is attenuated in IL-2 producing CD8 T cells

**Figure 5.**
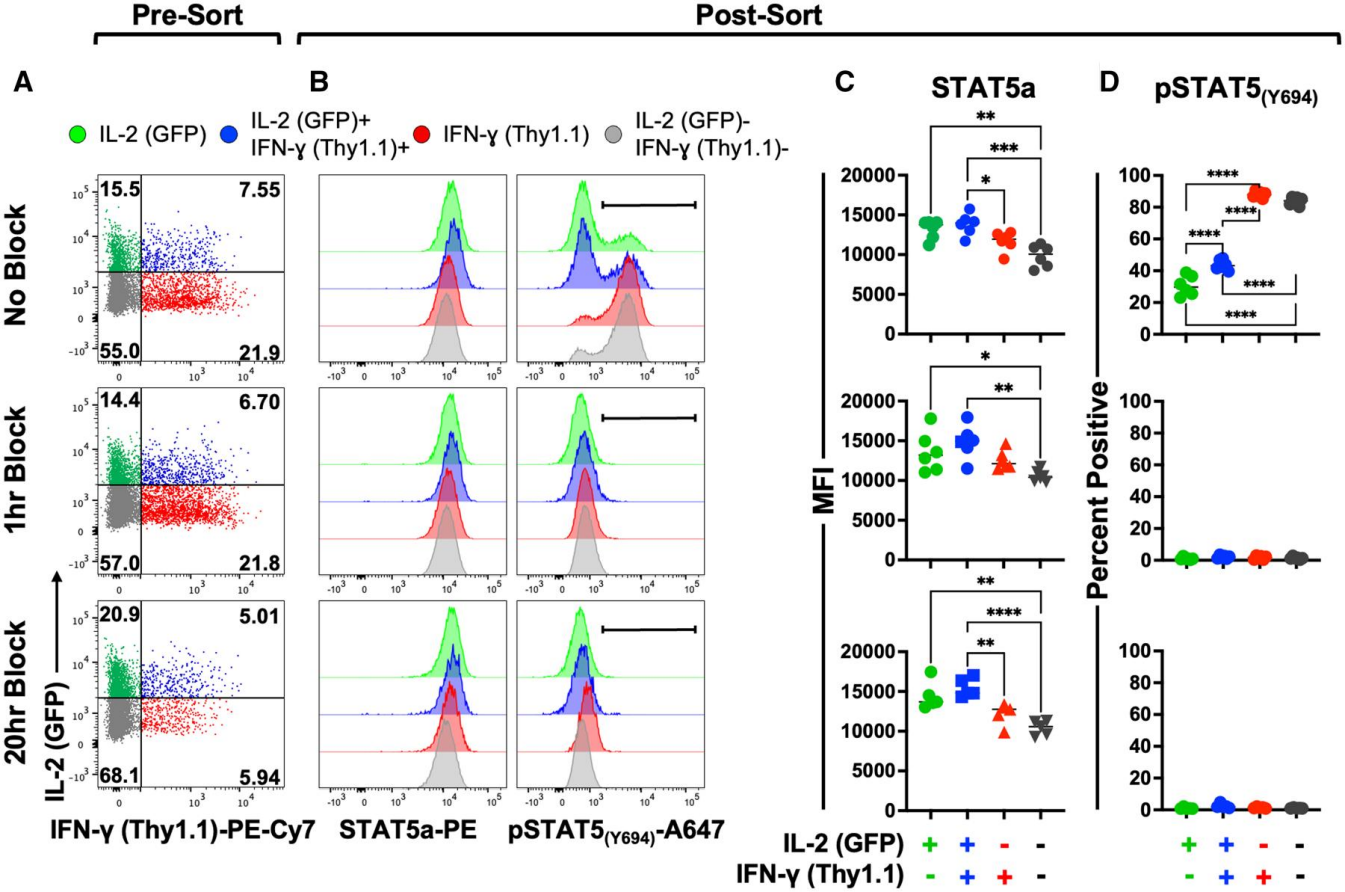
IL-2-dependent STAT5 phosphorylation is attenuated in IL-2 producing CD8 T cells. Naïve P14 cytokine reporter CD8 T cells were activated in vitro for 20 h with or without blocking of IL-2 signals for the last 1 h or entire duration of the stimulation period. (A) Representative flow cytometry plots show the expression of IL-2 (GFP) and IFN-γ (Thy1.1) by gated CD8 T cells prior to sorting. (B–D) The distinct cytokine producing subsets were sorted and the levels of STAT5a, and pSTAT5 (pY694) assessed. (B) Representative histograms, (C) composite results of the MFI of STAT5a staining, and (D) the percentages of pSTAT5-positive cells within each functionally distinct population. Significance was determined using one-way ANOVA. *P < 0.05, **P < 0.01, ***P < 0.001, and ****P < 0.0001. Representative or composite data are shown from 2 independent experiments analyzing a total of 3 male and 3 female mice.

Since both autocrine and paracrine activation by IL-2 have been implicated in effector and memory CD8 T cell differentiation,^13–17^ we assessed the phosphorylation (p) of STAT5, which conveys IL-2 signals (Fig. 5).^31–34^ Splenocytes from naïve P14 cytokine reporter mice were activated in vitro for 20 h and then IL-2 producing, IFN-γ producing, IL-2 and IFN-γ co-producing, or cytokine negative subsets sorted based on reporter expression (Fig. 5A, Fig. S2B). The levels of STAT5a and pSTAT5 in the sorted subsets was then determined (Fig. 5B–D). The subsets which manufactured IL-2 only or co-produced both IL-2 and IFN-γ expressed slightly higher levels of STAT5a (Fig. 5B and C). Whereas the vast majority of the CD8 T cells that were cytokine negative or only produced IFN-γ phosphorylated STAT5, markedly fewer CD8 T cells that only produced IL-2 or co-produced both IL-2 and IFN-γ were pSTAT5 positive (Fig. 5B, D). The observed phosphorylation of STAT5 was dependent on IL-2 as this was completely ablated by antibody-based blocking of IL-2 signals for either the last 1 hr (Fig. 5B–D middle panels) or entire duration of the 20 h stimulation period (Fig. 5B–D lower panels). Interestingly, the 20 h IL-2 blockade regimen slightly increased the fraction of IL-2 only producing CD8 T cells but restricted the development of the IFN-γ producing contingent (Fig. 5A). Thus, the early burst of IL-2 production by CD8 T cells is associated with significant reduction in IL-2-dependent STAT5 phosphorylation.

### Early functional diversification is associated with overlapping and distinct transcriptional changes

**Figure 6.**
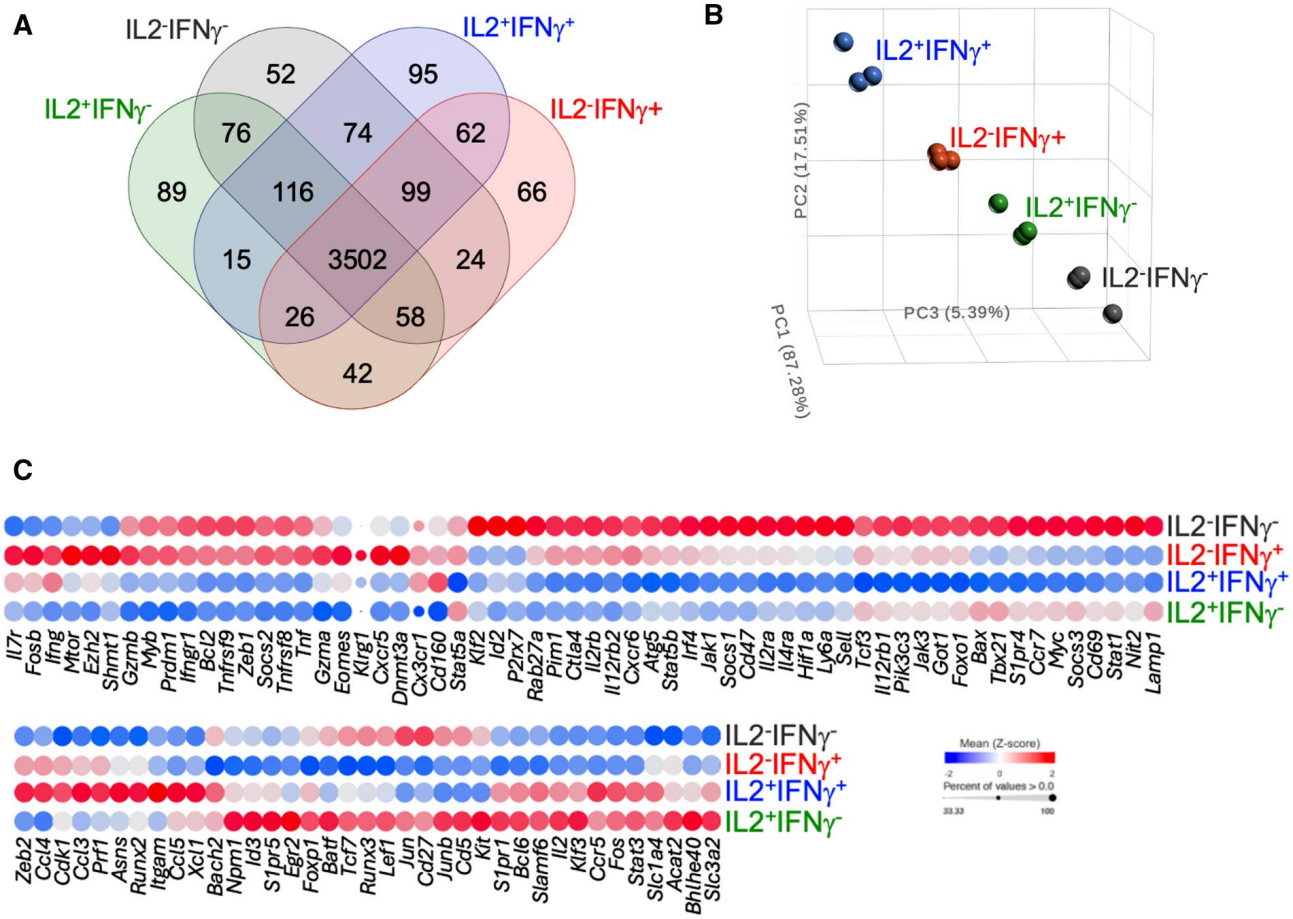
Early functional diversification is associated with overlapping and distinct transcriptional changes. RNA-sequencing was conducted to compare the transcriptome of naïve P14 CD8 T cells and the functionally distinct populations that manifest following stimulation with GP33 peptide for 20 h. (A) Venn diagram shows the numbers of overlapping and distinct differentially expressed genes by each functionally distinct subset when compared to naïve P14 CD8 T cells. (B) Principal component analyses focused on the genes that are differentially expressed among the four distinct sorted populations. (C) Bubble plot illustrates the relative expression of select effector and memory associated genes by the four functionally distinct populations. Composite analyses are shown from one female and two male mice.

We speculated that the early differences in manufacture of IL-2 and IFN-γ were underscored by divergent transcriptional profiles that may, in turn, influence the subsequent differentiation of the responding CD8 T cells. Therefore, we used the double cytokine reporter system to procure P14 CD69^+^ CD8^+^ IL-2^+^IFN-γ^-^, IL-2^+^IFN-γ^+^, IL-2^-^IFN-γ^+^, and IL-2^-^IFN-γ^-^ T cells at 20 hs following activation and conducted RNA-sequencing (Fig. 6, Fig. S2C). When compared with control naïve P14 CD8 T cells, a common core set of 3502 genes were differentially expressed by each functionally distinct subset. Nevertheless, each subset was also transcriptionally distinct, with varying degrees of transcriptional overlap between each population (Fig. 6A). We conducted a supervised principal component analysis focused on the genes that are differentially expressed by the four functionally distinct populations (Fig. 6B). This further demonstrated that even though the activated cells shared considerable overlap, each functionally discrete population was also transcriptionally distinct. We next scrutinized the expression of genes associated with effector and memory T cell formation, function, and maintenance^25–30^ (Fig. 6C). These analyses confirmed unique transcriptional aspects of each subset. Moreover, the differences in gene expression profiles, detectable by 20 h following activation, were suggestive of an early bias towards effector or memory development. For example, *Bcl6* was expressed at higher levels by IL-2^+^ cells, regardless of IFN-γ expression, whereas *Prdm1* was more elevated in IL-2^-^ subsets. Since *Bcl6* is associated with memory development and *Prdm1* influences effector formation,^35–38^ this suggests that preferred cell fates may be potentially forecasted prior to cell division by whether or not the responding cells undergo an initial round of IL-2 synthesis. This is further supported by the finding that the transcription factors *Bach2* and *Id3* were most prominently expressed in IL-2^+^, IFN-γ^−^ CD8 T cells, indicating that they may be poised to gain memory traits.^26^^,^^27^^,^^39–41^ Conversely, IL-2^-^IFN-γ^-^ CD8 T cells express relatively higher levels of *Id2,*^39^^,^^42^  *Irf4,*^43–45^  *Hif1α*^46–48^ and *Tbx21,*^49–51^ which are associated with effector formation. Although the results are indicative of emerging fate biases, certain patterns of expression do not neatly fit into effector or memory associated profiles. For example, the transcriptional regulators *Batf,*^52^^,^^53^  *Runx3,*^54–56^  *Lef1* and *Tcf7,*^57–59^ are relatively elevated in both the IL-2^+^IFN-γ^-^ and IL-2^-^IFN-γ^-^ subsets, suggesting that their fates are not cemented and likely evolve as they adapt to the ongoing antigenic, cytokine, nutrient, and cellular environment.

### Recently activated IL-2-producing and non-producing CD8 T cells are distinct following cell transfer

**Figure 7.**
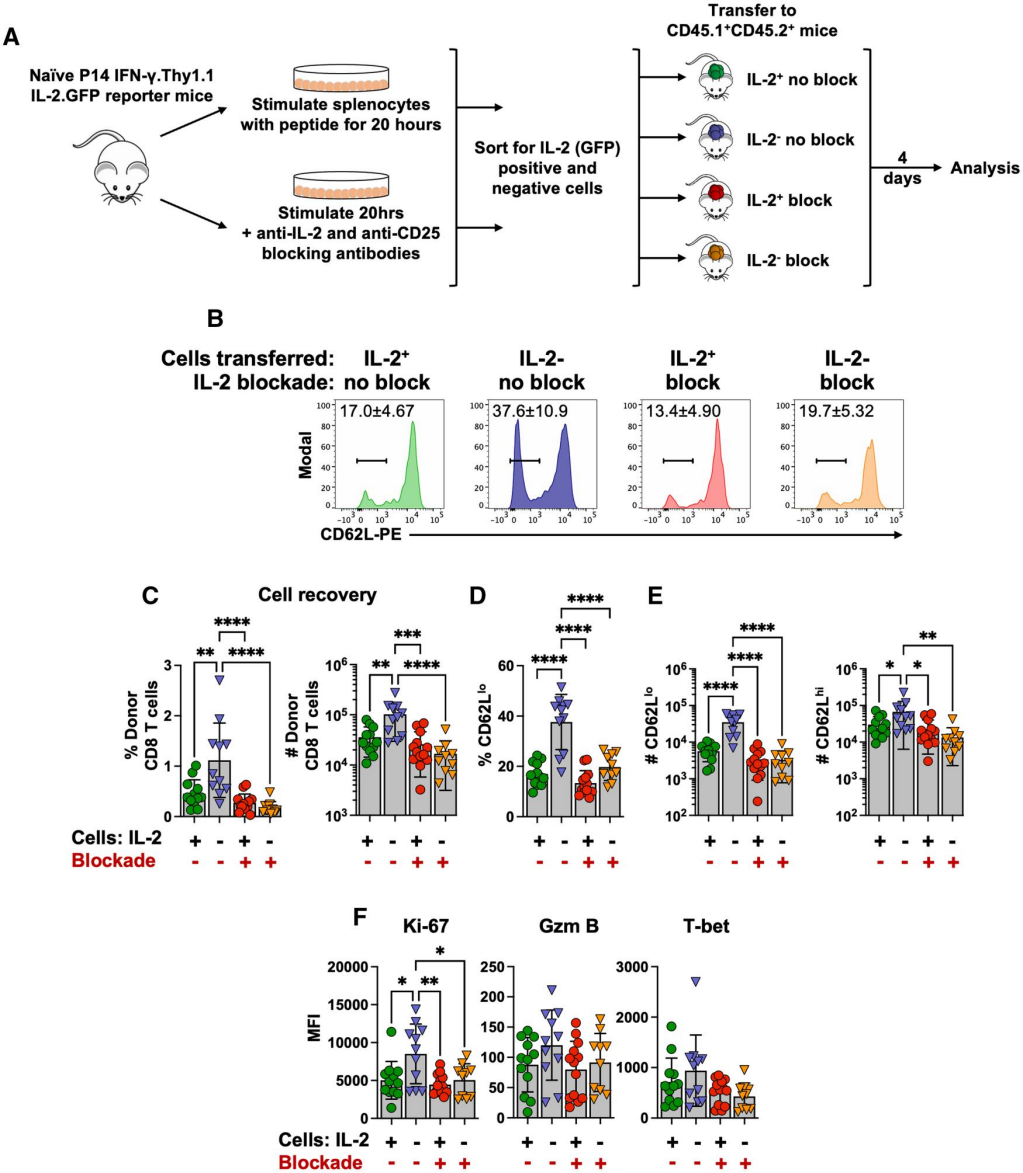
Recently activated IL-2-producing and non-producing CD8 T cells become phenotypically distinct following cell transfer. Splenocytes from naïve P14 TCR transgenic mice were stimulated for 20 h with the GP33 peptide antigen with or without the addition of IL-2 blocking antibodies and sorted based on IL-2 production. Normalized numbers of individual sorted populations (10^6^ cells) were then transferred into allelically distinct naïve recipients and analyzed four days post-transfer. (A) Schematic of experimental design. (B) Representative histograms show the levels of CD62L expression by the donor populations 4 d after transfer. Bar graphs show the cell recoveries (C), percentages of CD62^lo^ donor cells (D), the absolute numbers of CD62L^lo^ and CD62L^hi^ donor cells (E), as well as the MFI of Ki-67, granzyme B (Gzm B), and T-bet expression (F) by each donor cell subset at 4 days following cell transfer. Significance was determined using one-way ANOVA. Error bars show SD. *P < 0.05, **P < 0.01, ***P < 0.001, and ****P < 0.0001. Data are from 3 independent experiments analyzing a total of 10–13 male mice.

We next tested whether early differences in the induction of IL-2 and the receipt of IL-2 signals during the first 20 h of activation influenced the phenotype of the responding cells. To address this, splenocytes from naive P14 IFN-γ.Thy1.1 IL-2.GFP reporter mice were activated in vitro with the LCMV GP33 antigenic peptide. Parallel cultures were also set up in which IL-2 signals were blocked by the addition of anti-IL-2 and anti-CD25 antibodies. Following activation, non-IL-2 producing and IL-2-producing CD8 T cells from the control and blocked cultures were isolated by cell sorting and normalized numbers of each population were transferred into separate naive allelically marked recipients (Fig. 7A). The phenotypes and recoveries of the donor CD8 T cells were analyzed at 4 days following transfer (Fig. 7B–F). The IL-2-producing donor cells retained expression of CD62L, whereas a significant fraction of non-producing cells lost CD62L (Fig. 7B). Lower percentages and absolute numbers of IL-2-producing donor cell derivatives were recovered when compared with the non-IL-2 producing contingent (Fig. 7C), and the higher cell recovery of the non-IL-2 producing donor cells was ablated when IL-2 signals were blocked during the initial activation period. Although the IL-2-producing donor cells preferentially retained expression of CD62L, a significant percentage and number of the recovered non-IL-2 producing donor cells were CD62L^lo^ (Fig. 7B, D and E). Moreover, blockade of IL-2 during the 20 h activation period did not impact CD62L levels on the IL-2-producing contingent of cells recovered 4 d following transfer; however, it did prevent the loss of CD62L expression by the non-producing subset (Fig. 7B and D-E). The IL-2-producing CD8 T cells expressed lower levels of Ki67, by comparison with the non-producing cells, and this was consistent with a lower percentage and absolute number of IL-2-producing donor cells recovered from the recipients (Fig. 7C and F). The gain of Ki67 expression by the non-IL-2 producing cells was reversed by blocking IL-2 during the initial activation period, which is consistent with the non-IL-2 producing cells being permissive to IL-2 signals that influence their subsequent expansion and differentiation (Fig. 7F). Although not statistically significant, there was a trend that granzyme B and T-bet levels were higher in the recovered non-IL-2 producing donor cells, and this was reduced by blocking IL-2 signals at the time of activation (Fig. 7F). Thus, the manufacture of IL-2 by recently activated CD8 T cells, and IL-2 signaling to non-IL-2-producing cells, appear to shape their initial expansion and differentiation.

### IL-2-producing and non-producing CD8 T cells exhibit opposing early fate biases following infection

**Figure 8.**
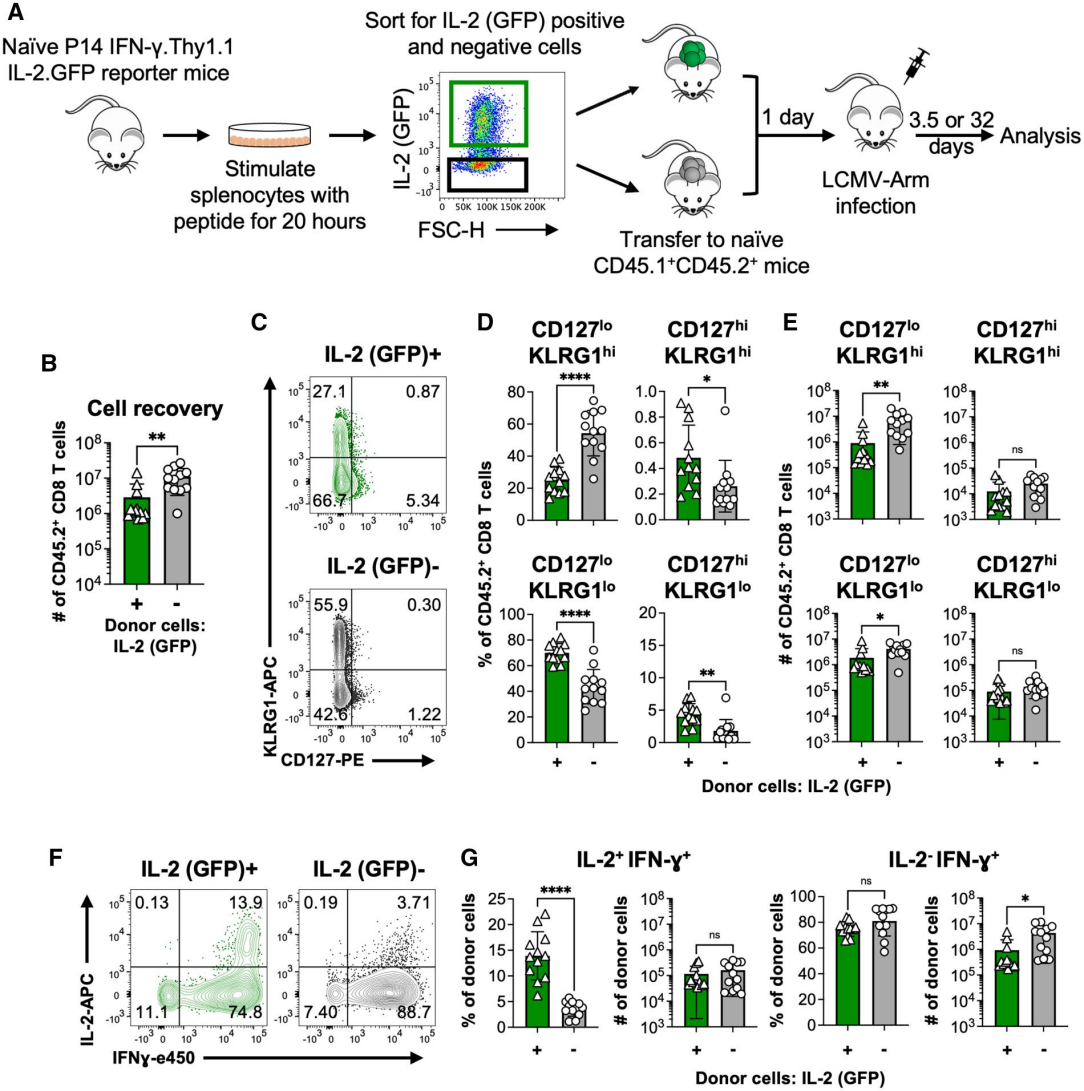
IL-2 producing and non-producing CD8 T cells exhibit an early fate bias following infection. Splenocytes from naïve P14 TCR transgenic IFN-γ.Thy1.1 IL-2.GFP reporter mice were stimulated for 20 h with the GP33 peptide and sorted based on IL-2 (GFP) production. Normalized numbers of individual sorted populations (2.5 × 10^5^ cells) were then transferred into allelically distinct naïve recipients which were infected one day later with LCMV. (A) Schematic of experimental design. (B) Recovery of IL-2^+^ and IL-2^-^ donor derived CD8 T cells at 3.5 d following infection. (C) Representative CD127 and KLRG1 cell staining profiles of the donor cell populations following infection; (D) frequencies and (E) numbers of recovered donor cells based on the patterns of CD127 and KLRG1 expression. (F) Representative staining profiles, (G) percentages and absolute numbers of recovered donor cells that are capable for producing IFN-γ and/or IL-2 at 3.5 d following infection. Bar graphs show means ± SD with significance calculated using an unpaired 2-tailed *t* test. *P < 0.05, **P < 0.01, ***P < 0.001, and ****P < 0.0001. Representative or cumulative results are shown from two independent experiments at each time point analyzing a total of 6 male and 6 female mice per group.

**Figure 9.**
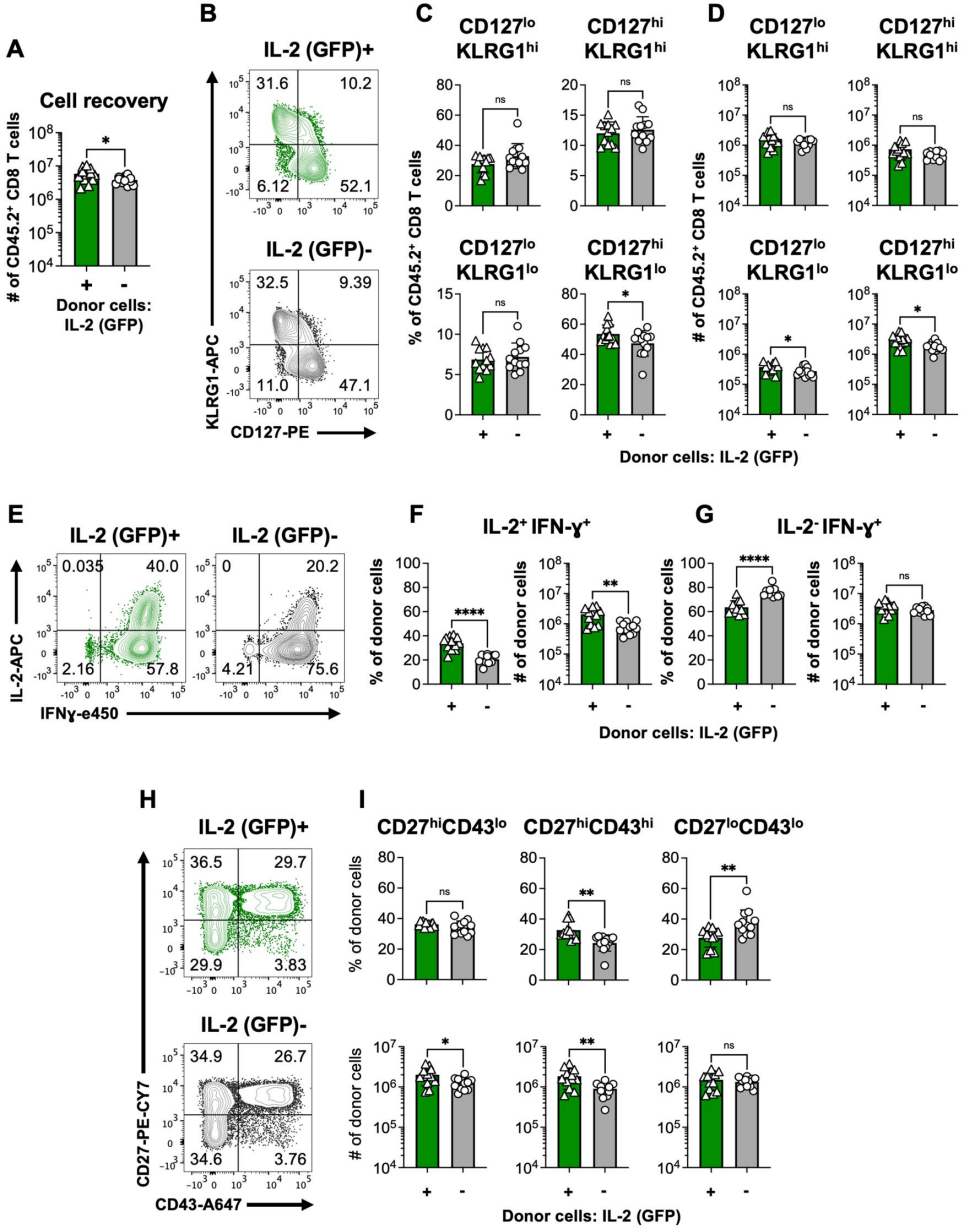
IL-2 producing and non-producing CD8 T cells attain memory properties. IL-2^+^ and IL-2^-^ donor cells were sorted at 20 h following activation. Allelically distinct naïve recipient mice were injected with 2.5 × 10^5^ cells of either population and infected 1 d later with LCMV, as in Fig. 8. Donor cells recovered from the spleens of the recipient mice were analyzed at 32 d post infection. (A) The numbers of each donor derived subset recovered at 32 d. (B) Representative staining profiles; (C) frequencies and (D) numbers of recovered donor cells based upon their expression of CD127 and KLRG1. (E) Representative staining profiles, percentages and absolute numbers of (F) IL-2 and IFN-γ co-producing or (G) IFN-γ only producing donor cells. (H) Representative staining profiles; (I) frequencies and numbers of recovered donor cells based upon their expression of CD43 and CD27. Bar graphs show means ± SD with significance calculated using an unpaired 2-tailed *t* test. *P < 0.05, **P < 0.01, ***P < 0.001, and ****P < 0.0001. Representative or cumulative results are shown from two independent experiments at each time point analyzing a total of 6 male and 6 female mice per group.

To further investigate the role of early intrinsic IL-2 production in shaping CD8 T cell fates, IL-2-producing and non-producing CD8 T cells were isolated at 20 h following activation and transferred into allelically marked recipients that were infected with LCMV-Armstrong (acute strain) one day later (Fig. 8A). The properties of the donor cells were analyzed at 3.5 (Fig. 8) and 32 (Fig. 9) days post infection. At day 3.5 following infection 2.86 × 10^6^±3.98 × 10^6^ IL-2 producing donor cells and 1.11 × 10^7^±7.81 × 10^6^ non-IL-2 producing donor cells were recovered from the recipients (Fig. 8B). This greater clonal expansion of the non-IL-2-producing subset following infection was consistent with the larger cell recoveries detected in naive recipients (Fig. 7C). At this early time point almost all of the donor cells had downregulated CD127 expression in the infected recipients; nevertheless, a slightly higher fraction of the IL-2 producing donor cells displayed a CD127^hi^KLRG1^lo^ memory precursor effector cell (MPEC)^50^^,^^60^^,^^61^ phenotype when compared with their non-IL-2 producing counterparts (Fig. 8C–E). More strikingly, a greater proportion and absolute number of non-IL-2 producing donor cells gained a CD127^lo^KLRG1^hi^ short-lived effector (SLEC) phenotype^50^^,^^60^^,^^61^ (Fig. 8C–E). We also evaluated the capacity of the recovered donor cells to produce IL-2. The majority of the donor cells that produced IL-2 at the time of transfer lost the ability to manufacture IL-2 by 3.5 d after infection, whereas a fraction of the non-IL-2-producing donor cells gained the ability to synthesize IL-2 following transfer. Comparison of the two donor subsets did, however, show that a greater proportion of the IL-2-producing donor cells retained the ability to produce IL-2 (Fig. 8F and G). Thus, very early differences in the ability of CD8 T cells to produce IL-2 was associated with phenotypic differences detectable within 3.5 d following infection, and a clear-cut bias in the gain of a SLEC phenotype by the non-IL-2-producing donor cells.

By day 32 following infection 5.85 × 10^6^±2.65 × 10^6^ and 3.83 × 10^6^±9.85 × 10^5^ IL-2-producing and non-producing donor cells were recovered, respectively (Fig. 9A). By this time point, in comparison with the derivatives of the non-IL-2-producing donor cells, a slightly higher proportion and ∼1.7 fold greater number of the IL-2-producing donor cells adopted a CD127^hi^KLRG1^lo^ canonical memory phenotype (Fig. 9B–D).^50^^,^^60^^,^^61^ Conversely, similar abundances of residual CD127^lo^KLRG1^hi^ effector phenotype cells were present in both donor cell pools (Fig. 9B-D). Consistent with the day 3.5 results, a greater fraction of donor cells that produced IL-2 at the time of transfer could manufacture IL-2 at day 32, and approximately one fifth of the non-IL-2 producing donor cells could also now synthesize IL-2 (Fig. 9E–G). CD27 and CD43(1B11) expression delineate 3 populations of memory CD8 T cells, which differ in their abilities to accumulate following secondary challenge and confer immunological protection.^62^^,^^63^ By day 32, a slightly higher fraction of the IL-2-producing donor cells attained a CD27^hi^CD43^hi^ phenotype. By contrast, a greater proportion of the non-IL-2 producing donor cells were CD27^lo^CD43^lo^ (Fig. 9H and I), which may suggest superior protective efficacy.^63^ Collectively, these findings show that the very early burst of IL-2 production, which occurs prior to the first cell division following activation, is associated with reduced IL-2-dependent STAT5 phosphorylation, unique transcriptional profiles, and a restricted ability to gain early effector traits. Nevertheless, these differences are not absolute. Thus, at the population level, this manifests as a phenotypic bias rather than outright imprinting.

## Discussion

Our findings demonstrate that as naïve CD8 T cells become activated by their cognate antigen, they undergo a rapid, heterogenous, and dynamic burst of IL-2 and IFN-γ synthesis prior to cell division. Although the distinct IL-2^+^, IL-2^+^IFN-γ^+^, IFN-γ^+^, and IL-2^-^ IFN-γ^-^ subsets express similar surface levels of the IL-2 receptor complex, they differ in their ability to elaborate IL-2-dependent STAT5 signaling. The observed functional diversity is further associated with both overlapping and distinct gene expression profiles. These emerging transcriptional landscapes coupled with differences in the sensitivity to cytokine signals likely influence the developmental preferences of the nascent response. Nevertheless, at this primordial stage, these biochemical and genetic distinctions likely bias rather than indelibly etch the cells subsequent capacity to form effector and memory subsets.

The early functional diversification appears to be a common immunological theme as it manifests with every specificity of CD8 T cells checked. Intriguingly, the uniform and controlled activation of naïve T cells, which express clonally identical TCRs, results in both the heterogeneous induction of cytokine production and unique transcriptional profiles. These early differences may occur because individual T cells do not necessarily encounter or engage presented antigen in a consistent or synchronized manner. It is also plausible that heterogeneity within the naïve CD8 T cells pool contributes to unevenness in their activation and differentiation.^64^^,^^65^ For example, by comparison with mature naïve CD8 T cells, recent thymic emigrants have been shown to elaborate increased TCR signaling as well as form fewer memory precursor phenotype cells following activation.^66^^,^^67^ Additionally, variances in CD5 levels are associated with differences in the responsiveness to cytokine signals, including IL-2 and IL-7,^68^ and it is notable that distinct levels of CD5 transcripts are present in the subsets analyzed in this study. Nevertheless, even though naïve T cells may exhibit some level of heterogeneity, prior studies have shown that the progeny of a single naïve T cell can go on to differentiate into multiple effector and memory subsets.^69^^,^^70^ Thus, the future fate of a naïve CD8 T cell is not necessarily predetermined, but instead influenced by the collective cues encountered during and following activation.

A key finding from this study is that the rapid induction of IL-2 production by recently activated naïve CD8 T cells is associated with diminished IL-2-dependent STAT5 phosphorylation. This has potential implications for effector and memory T cell formation as prolonged IL-2 signaling during the induction phase of the response promotes effector phenotypes.^9^^,^^11^^,^^12^ This is consistent with the results of adoptive transfer and tracking of IL-2-producing and non-producing CD8 T cells reported here, which shows that the non-IL-2 producing subsets, which exhibit increased STAT5 activation, are prone to initially gain short-lived effector properties. Dichotomies between the synthesis of IL-2 and STAT5 phosphorylation have been previously reported for both CD4 and CD8 T cells.^17^^,^^18^^,^^24^^,^^71^^,^^72^ In the case of CD4 T cells, restricted STAT5 activation by IL-2 producers is associated with preferential T follicular helper differentiation.^18^ For CD8 T cells, IL-2 synthesis and phosphorylated STAT5 are predominately detected in mutually exclusive populations during the effector phase of the response. In this case, the contingent of effector CD8 T cells that do not manufacture IL-2 but are permissive to paracrine IL-2 signals, more stringently display an effector associated phenotype but fail to protect against a chronic viral challenge. By contrast, the IL-2 producing effector populations preferentially display stem-like memory traits, are less prone to exhaustion, and mount superior recall responses.^17^

Although the chains of the IL-2 receptor are similarly expressed at the surface of each functional subset, IL-2 producing CD8 T cells have lower levels of *Il2ra* (CD25) and *Il2rb* (CD122) transcripts. This discrepancy between mRNA and surface protein levels may be a consequence of enhanced IL-2-signaling by the non-IL-2-producing subsets, which can result in internalization of the IL-2 bound receptor complex.^73^ Moreover, the IL-2^+^ IFN-γ ^+^ CD8 T cells transcriptionally express the lowest relative levels of *Stat5a* and *Stat5b*, whereas they have the most abundant amounts of STAT5a protein. This discordance may be due to multiple reasons. First, mRNA levels do not necessarily directly correlate with protein levels. Second, phosphorylation of STAT5 has been reported to decrease the levels of nucleophosmin-1, which is consistent with the lower *Npm1* transcript levels in the non-IL-2 producing CD8 T cells. Notably, diminutions in nucleophosmin-1 can reduce the abundance of STAT5.^74^

The underlying factors responsible for the inverse correlation between the cell-intrinsic manufacture of IL-2 and the extent of IL-2-dependent STAT5 activation remain unclear. *Socs1*, *Socs2,* and *Socs3,* as well as *Pim1,* have all been reported to regulate STAT5 signaling; however, their transcript levels are highest in the activated IL-2^-^ IFN-γ^-^ CD8 T cells.^33^^,^^75–77^ Thus, these may act to counter-regulate STAT5 signaling in the non-IL-2 producing subsets, but distinct mechanisms likely operate to restrict responses in the IL-2-producers. Interestingly, it has been shown that the IL-2 receptor complex preassembles in the endoplasmic reticulum and Golgi, and can signal intracellularly if IL-2 is co-produced.^78^ Therefore, it is possible that the IL-2 producing subsets receive STAT5-independent autocrine signals, which influence their early transcriptional profiles but that these molecular instructions are insufficient or inappropriate for driving the rapid early differentiation of effector cells. Nevertheless, this is speculative and warrants further study.

We have shown heterogeneity in the patterns of IL-2 and IFN-γ synthesis following the activation of naïve CD8 T cells but before they undergo their first cell division. By harnessing the cytokine reporter platform, we were able to separate and track these functionally distinct subsets and demonstrate that the non-IL-2 producing subsets are favored to adopt an effector-like phenotype. This was indicated following transfer into naïve recipients as well as 3.5 d after LCMV infection. This apparent biasing of the initial developmental trajectory of CD8 T cells is also consistent with RNA-sequencing analyses conducted at 20 h following activation. There is a substantial overlap in the transcriptional attributes for every subset, independent of whether they synthesize IFN-γ or IL-2, as compared to naïve CD8 T cells. Conversely, each of the four subsets analyzed is also transcriptionally distinct, with divergent profiles that suggest an early bias towards effector or memory differentiation trajectories. At this early stage, however, the developmental fates of the responding cells are unlikely to be fully imprinted as transcriptional landscapes continue to evolve as the response progresses, they undergo division, and additional signals are received.^25^^,^^27^^,^^79–81^

Although we detect a propensity of non-IL-2 producing CD8 T cells to initially attain an effector-like state, by 32 d following priming the progeny of both IL-2-producing and non-producing CD8 T cells are more similar, albeit not identical. At this stage many of the donor cells that did not manufacture IL-2 initially gained a canonical CD127^hi^ KLRG-1^lo^ memory phenotype and approximately one fifth of these cells could produce detectable levels of IL-2 following stimulation. Thus, even though the non-IL-2 producing population is initially biased to attain a short-lived effector phenotype, sufficient numbers of these cells are capable of gaining memory properties. Nevertheless, by day 32 following transfer marginal but statistically significant differences in the fraction of IL-2-producting and non-producing donor cell derivatives that are CD127^hi^ KLRG1^lo^, IL-2 producing, and distinguishable by CD27 and CD43 (1B11 isotype) expression are detectable. Taken together, the non-IL-2 producers appear to be biased to gain effector traits but can form a substantial memory pool. By contrast, the initial induction of IL-2 synthesis results in a weaker effector burst but allows memory formation. Thus, CD8 T cell differentiation into effector and memory subsets may become biased during the initial 20 to 24 h activation period, when IL-2 and IFN-γ are first induced and as marked changes in gene expression are occurring. Nevertheless, the fates are not fully preset at this stage and are further steered by the antigenic, cytokine, and nutrient signals that are encountered as the response continues. In future studies it will be of interest to determine whether there are underlying differences in the epigenetic landscape and transcriptional profiles of the subsets that differentiate from these early activated populations, as well as investigate the parameters that direct the differentiation of these distinct subsets following the initial stimulation window.

## Supplementary Material

vkaf239_Supplementary_Data

## Data Availability

The RNA-sequencing data are deposited in the Gene Expression Omnibus under accession number GSE294688. Other data are available upon request without restriction, and are in the process of being deposit to a publicly available repository.
