# Supplementary material for: An early burst of cytokine production before the first cell division influences CD8 T cell differentiation
Source: J Immunol. 2025 Sep 16;215(1):vkaf239. doi: 10.1093/jimmun/vkaf239 (PMC12936695; doi:10.1093/jimmun/vkaf239)
Supplement: vkaf239_Supplementary_Data [file vkaf239_supplementary_data.pdf]

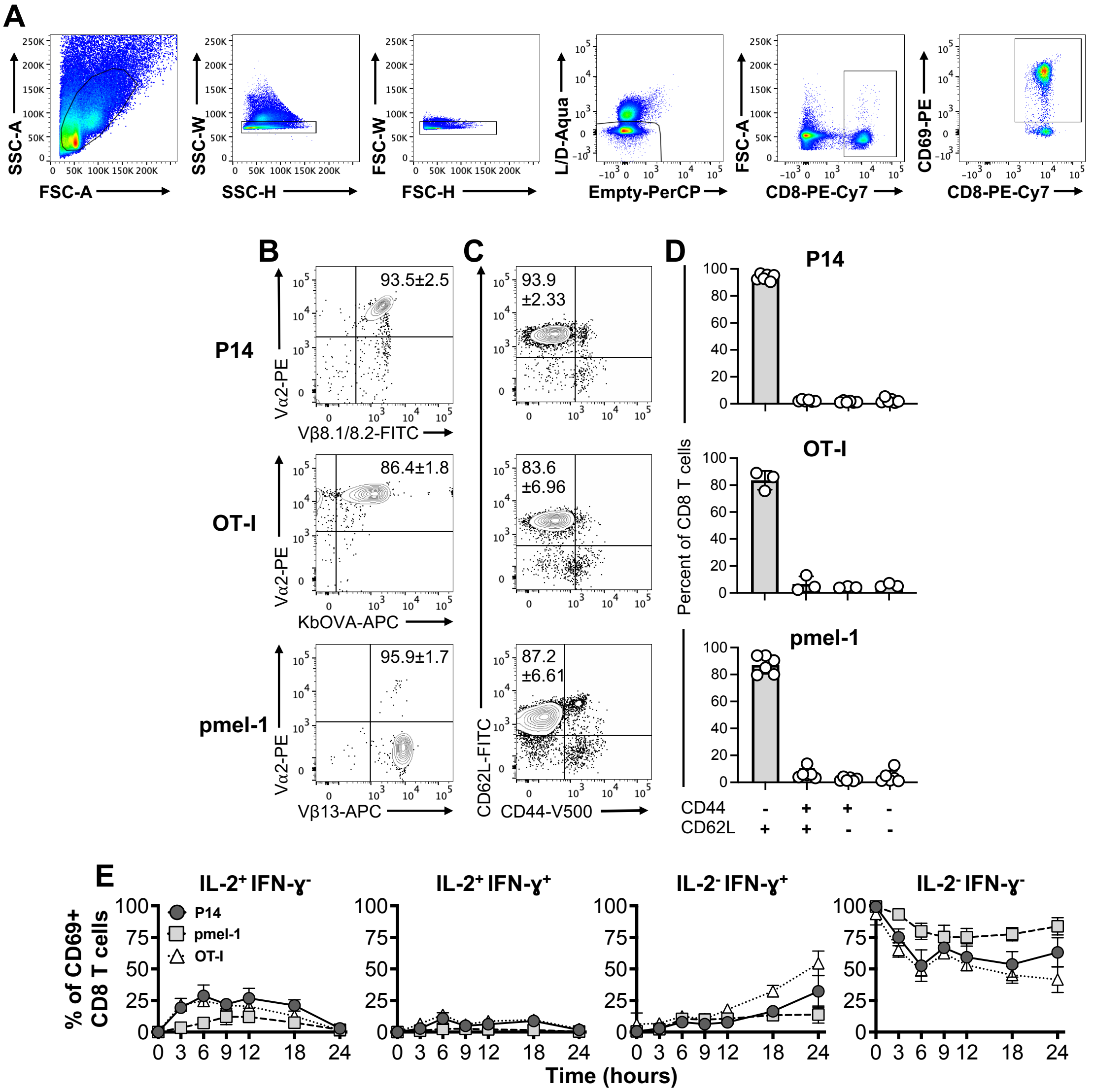

Figure S1. Supplementary material related to figure 1. (A) The flow cytometry gating strategy used for figure 1. (B) Flow cytometric analyses of penetrance of TCR transgene expression by the P14, OT-I, and pmel-1 CD8 T cells. (C,D) Representative flow cytometry plots and composite results analyzing CD44 and CD62L expression by CD8+ splenocytes from P14, OT-I, and pmel-1 mice. (E) Comparisons of the percentages of IL-2^{+} IFN-γ^{-}, IL-2^{+} IFN-γ^{+}, IL-2^{-} IFN-γ^{+}, and IL-2^{-} IFN- γ^{-} CD69^{+} CD8 T cells for each TCR transgenic population over a 24 hr activation period. In B-D representative or composite results are shown from 3 male and 2 female P14 mice as well as a pool of cells from 2 male P14 mice; 1 male and 1 female OT-I mouse as well as a pool from 2 male OT-I mice; and 3 male and 2 female pmel-1 mice as well as a pool from 2 male pmel-1 mice. Findings in E are derived from the results shown in main Figure 1B and their statistical analyses are presented in Table S1.

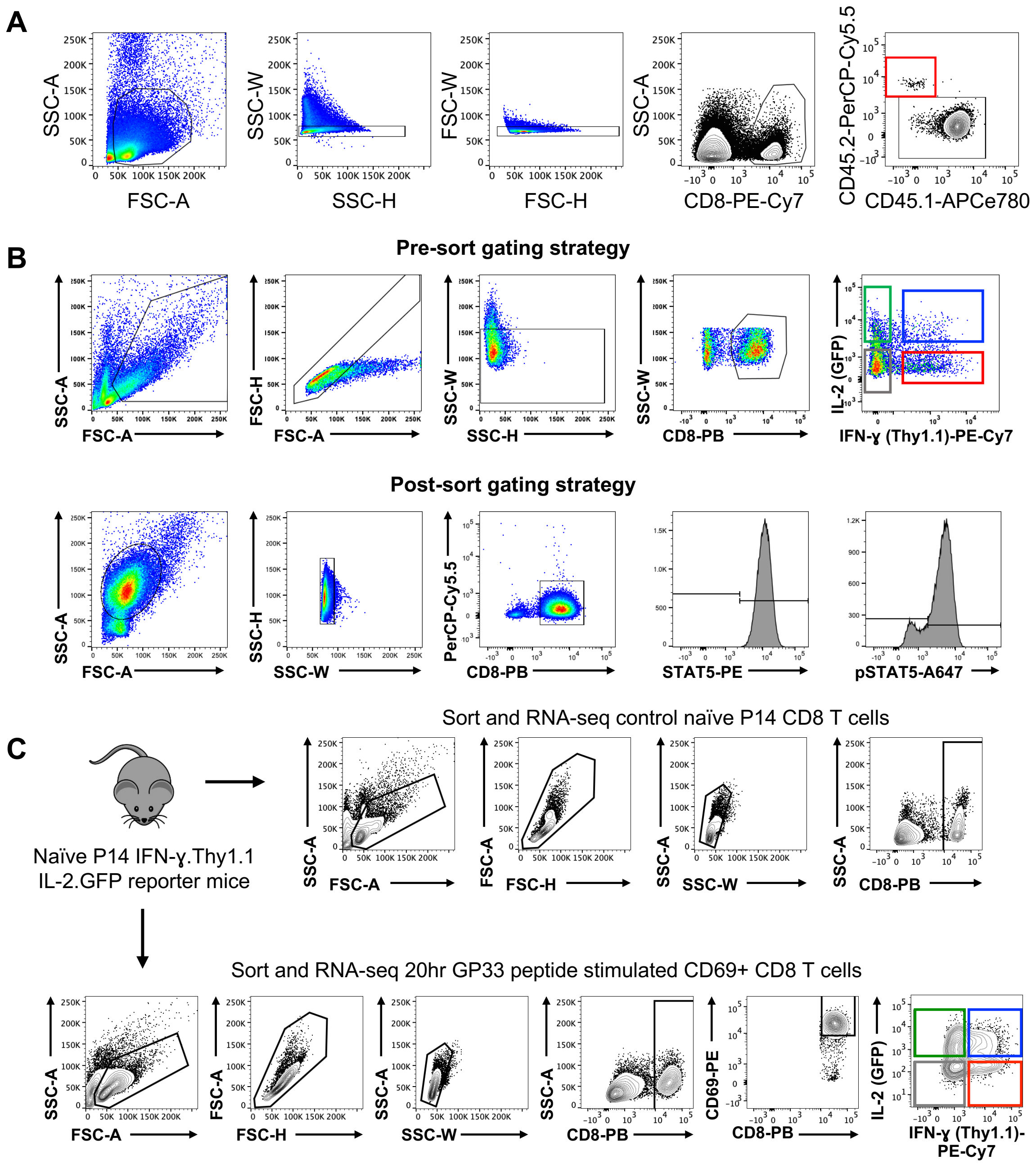

Figure S2. Representative gating strategies for the findings presented in main Figures 2 (A), 5 (B), and 6 (C).

Table S1. Statistical analyses of the results shown in Figure S1E

Time[a]	Comparison[b]	IFN-γ^{-}IL-2[c]	IFN-γ^{+}IL-2^{+}	IFN-γ^{+}IL-2^{-}	IFN-γ^{-}IL-2^{-}
3hr	P14 vs. pmel-1	<0.0001	ns	ns	<0.01
	P14 vs. OT-I	ns	ns	ns	ns
	pmel-1 vs. OT-I	<0.0001	<0.01	ns	<0.0001
6hr	P14 vs. pmel-1	<0.0001	<0.0001	ns	<0.0001
	P14 vs. OT-I	ns	ns	ns	ns
	pmel-1 vs. OT-I	<0.0001	<0.0001	ns	<0.0001
9hr	P14 vs. pmel-1	<0.01	ns	ns	ns
	P14 vs. OT-I	ns	ns	ns	ns
	pmel-1 vs. OT-I	<0.05	ns	ns	ns
12hr	P14 vs. pmel-1	<0.0001	<0.01	ns	<0.01
	P14 vs. OT-I	ns	ns	<0.05	ns
	pmel-1 vs. OT-I	<0.05	<0.0001	ns	<0.001
18hr	P14 vs. pmel-1	<0.0001	<0.0001	ns	<0.0001
	P14 vs. OT-I	<0.01	ns	<0.0001	ns
	pmel-1 vs. OT-I	ns	<0.0001	<0.0001	<0.0001
24hr	P14 vs. pmel-1	ns	ns	<0.0001	<0.0001
	P14 vs. OT-I	ns	ns	<0.0001	<0.001
	pmel-1 vs. OT-I	ns	ns	<0.0001	<0.0001

[a] Number of hours that the cells were activated for prior to analyses.
[b] Pairwise comparisons of the percentages of TCR transgenic CD8 T cells with the indicated functional attributes were done by 2-way ANOVA with Tukey comparison testing.
[c] P values; ns, not significant (>0.05).
